# A *p53-Pax2* Pathway in Kidney Development: Implications for Nephrogenesis

**DOI:** 10.1371/journal.pone.0044869

**Published:** 2012-09-12

**Authors:** Zubaida Saifudeen, Jiao Liu, Susana Dipp, Xiao Yao, Yuwen Li, Nathaniel McLaughlin, Karam Aboudehen, Samir S. El-Dahr

**Affiliations:** 1 Section of Pediatric Nephrology, Department of Pediatrics, Tulane University Health Sciences Center, New Orleans, Louisiana, United States of America; 2 The Hypertension and Renal Center of Excellence, Tulane University Health Sciences Center, New Orleans, Louisiana, United States of America; University of Texas MD Anderson Cancer Center, United States of America

## Abstract

Congenital reduction in nephron number (renal hypoplasia) is a predisposing factor for chronic kidney disease and hypertension. Despite identification of specific genes and pathways in nephrogenesis, determinants of final nephron endowment are poorly understood. Here, we report that mice with germ-line *p53* deletion (*p53^−/−^*) manifest renal hypoplasia; the phenotype can be recapitulated by conditional deletion of *p53* from renal progenitors in the cap mesenchyme (CM^p*53−/−*^). Mice or humans with germ-line heterozygous mutations in *Pax2* exhibit renal hypoplasia. Since both transcription factors are developmentally expressed in the metanephros, we tested the hypothesis that *p53* and *Pax2* cooperate in nephrogenesis. In this study, we provide evidence for the presence of genetic epistasis between *p53* and *Pax2*: a*) p53^−/−^* and CM*^p53−/−^*embryos express lower Pax2 mRNA and protein in nephron progenitors than their wild-type littermates; *b)* ChIP-Seq identified peaks of p53 occupancy in chromatin regions of the *Pax2* promoter and gene in embryonic kidneys; *c)* p53 binding to *Pax2* gene is significantly more enriched in Pax2 -expressing than non-expressing metanephric mesenchyme cells; *d)* in transient transfection assays, *Pax2* promoter activity is stimulated by wild-type p53 and inhibited by a dominant negative mutant p53; *e)* p53 knockdown in cultured metanephric mesenchyme cells down-regulates endogenous *Pax2* expression; *f)* reduction of *p53* gene dosage worsens the renal hypoplasia in *Pax2* ^+/−^ mice. Bioinformatics identified a set of developmental renal genes likely to be co-regulated by p53 and Pax2. We propose that the cross-talk between *p53* and *Pax2* provides a transcriptional platform that promotes nephrogenesis, thus contributing to nephron endowment.

## Introduction

Mammalian kidney development is dependent on reciprocal inductive interactions between the metanephric mesenchyme and the ureteric bud (reviewed in [1]). Nephrons arise exclusively from the nephron progenitor cell population that is adjacent to the ureteric bud, and is referred to as the cap mesenchyme (CM) [2], [3]. Conversion of the progenitor CM cells to nephron epithelia requires co-ordinated expression of key transcriptional regulators that drive expression of distinct gene targets [4]–[6]. The CM expresses key transcription regulators that include *Six2, Pax2, Eya1, Sall1, Wt1, Meox*, and *Cited1* ([1], [7], reviewed in [8]). These genes, either individually or combinatorially, drive cell-survival, self-renewal, differentiation and nephrogenesis.

The paired box (*Pax*) gene family of transcription factors consists of nine members that are grouped into four classes based on their DNA binding specificities and developmental expression [9], [10]. Of the *Pax2/5/8* sub-group, expression of *Pax2/8* is essential and sufficient to induce nephric lineage and nephric duct morphogenesis, and both genes are required for nephron differentiation [11], [12]. *Pax2^−/−^* mice exhibit anomalous development of the midbrain, the cerebellum [13] and the optic nerve [14] and renal agenesis [15]. Pax2 is a known determinant of ureteric bud (UB) branching and nephron number and *Pax2* haplo-insufficiency in mice or humans are associated with renal hypodysplasia [16], [17]. Pax2, in concert with Hox11 and Eya1, is required for expression of *Six2* and *GDNF* [18], [19], essential regulators of nephron progenitor cell population and branching morphogenesis, respectively. *Pax2*-null mice lack *GDNF* expression and have decreased *Six2* expression [15], [20]. *Wnt4* is another important Pax2 target in the developing nephron. Decreased *Wnt4* expression was demonstrated *in vivo* in hypoplastic kidneys of heterozygous *Pax2* mutant mice [21].

The role of p53 in maintaining genomic stability is well-documented. In the absence of stress, p53 levels in adult cells are low. Stabilization and activation occur upon genotoxic or oncogenic stress, resulting in a transcriptional program that activates cell cycle arrest, apoptosis or senescence [22]–[24]. Induction of differentiation programs is also viewed as a tumor suppressor function of p53 since this results in removal of the cell from the proliferative pool [22]. p53 represses expression of pluripotency genes such as *Nanog* in mESCs thereby promoting their differentiation and preventing dedifferentiation of induced progenitors to pluripotent cells [25]. With respect to this function, *p53* inactivation results in loss of asymmetric cell division of stem cells resulting in only self-renewing symmetric cell division generating more stem cells [26], [27]. Thus, p53 plays a key role in cell-fate determination. In addition to these well-characterized roles, emerging evidence implicates p53 in tumor suppression by regulating genes in metabolic pathways, e.g., oxidative and glycolytic pathways for energy generation and glucose homeostasis [28] and anti-angiogenic pathways [29], [30].

*p53* expression is ubiquitously high during early embryonic life [31]. After mid-gestation, *p53* expression is tissue-specific and higher in differentiating tissues. Although *p53*-null mice develop tumors early (∼4–6 mo), they are viable and show genetic background dependent developmental abnormalities, such as defects in spermatogenesis, severe ocular abnormalities, and ∼20% female embryonic lethality due to exencephaly [32]–[37]. Developmental abnormalities with incomplete penetrance in mice are in contrast to embryonic lethality observed in Xenopus embryos upon p53 depletion [38], [39]. Compensation by p53 family members, p63 and p73, that are present in early mouse embryo has been suggested as a possible reason for the non-lethal phenotype [40]. We have described duplex ureter formation and renal hypoplasia in *p53^−/−^* mice [41], as well as impaired terminal differentiation of renal epithelia [42]. *p53* deletion also impairs differentiation of skeletal muscle cells, various hematopoietic cell-lines, thyroid cells, keratinocytes, oligodendrocytes [22], [43], [44], neuronal maturation, axon outgrowth and regeneration [45]. In contrast, excess p53 activity, achieved by deletion of its negative regulator MDM2, results in embryonic death at peri-implantation stage that can be rescued by *p53* deletion [46]. We recently showed that conditional *Mdm2* deletion in the nephric duct lineage derived ureteric epithelium results in severe hypodysplastic kidneys as a result of excessive apoptosis, decreased proliferation of the ureteric epithelium, and repression of the nephron inducer Wnt9b [47]. Inhibition of UB branching and concomitant nephrogenesis defects were rescued by genetic depletion of p53.

Like Pax2, p53 is expressed in the intermediate mesoderm and urogenital ridge and later in development in the mesonephros and metanephros [11], [41]. In this study, we demonstrate that *p53* and *Pax2* are epistatic and that p53 is an endogenous activator of *Pax2* gene expression during kidney development. These findings led us to propose that down-regulation of Pax2 contributes to renal hypoplasia in *p53*-null embryos.

## Results

### Nephron Deficit in Germ-line *p53^−/−^* Mouse Kidneys is Recapitulated by Conditional *p53* Deletion from *Six2+* Cap Mesenchyme

**Figure 1:**
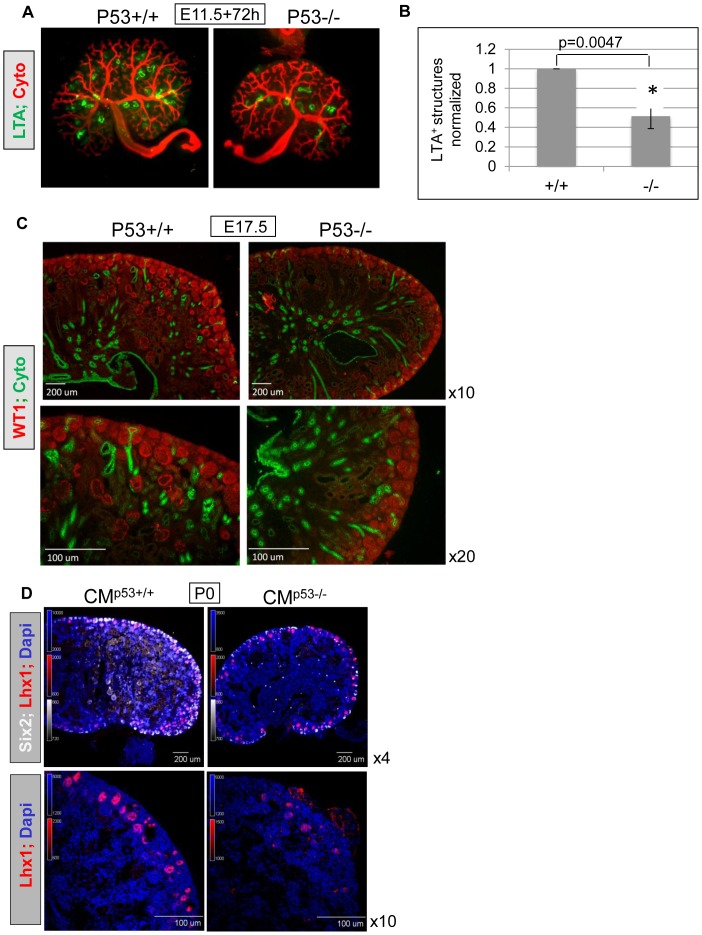
Nephron deficit in germ-line *p53^−/−^* and CM*^p53−/−^* kidneys. **A, B**) *p53^−/−^* kidneys exhibit 50% fewer and less complex LTA-positive proximal tubules. Metanephroi were harvested at E11.5, cultured on trans-well filters for 72 h and stained for cytokeratin and LTA. LTA counts were averaged from kidneys collected from at least 4 embryos. Mutant kidneys exhibit significantly fewer LTA+ structures than wild-type kidneys (p<0.005). **C)** E17.5 kidneys were harvested from embryos from *p53^+/−^* crosses, formalin-fixed and sectioned for immunostaining (Methods). Sections were stained with WT1 and cytokeratin antibodies. *P53^−/−^* kidneys show paucity of WT1 stained nephrons. **D**) Kidneys from mice with conditional *p53* deletion from *Six2+* cap mesenchyme (CM*^p53−/−^*). Hypoplasia persists post-natally, shown in P0 kidneys, top panels at 4x. Bottom panels (x10) show fewer Lhx1-positive nascent nephrons (red). At P0, 6/8 (75%) examined CM*^p53−/−^* kidneys were hypoplastic compared to wild-type littermate kidneys (n = 8).

We previously described a defect in UB branching morphogenesis in *p53^−/−^* mice [41]. Additional characterization of *p53^−/−^* kidneys revealed 50% fewer and less complex LTA-positive proximal tubules in E11.5 kidneys cultured for 72 h (Fig. 1A, B). Mutant kidneys exhibit significantly fewer LTA+ structures than wild-type kidneys (p<0.005). WT1 staining of E17.5 kidney sections showed fewer immature nephrons in the nephrogenic zone as well as a reduction of mature glomeruli in the inner cortex (Fig. 1C). Notably, the presence of renal hypoplasia and fewer nephrons in *p53^−/−^* metanephroi is recapitulated in mice with conditional *p53* deletion from *Six2+* **c**ap **m**esenchyme (CM*^p53−/−^*) (Fig. 1D), which are the nephron progenitor cells, suggesting an autonomous role for p53 in the CM in nephron formation. At P0, 6/8 (75%) examined CM*^p53−/−^* kidneys were hypoplastic compared to wild-type littermate kidneys (n = 8) (Fig. 1D). Persistence of hypoplasia post-natally suggests that the phenotype cannot be attributed simply to developmental delay. Lhx1 staining marks the differentiating nascent nephron population. Note fewer generations of Lhx1+ nascent nephrons in CM*^p53−/−^* kidneys (Fig. 1D, x10 images). Results of microarray analysis revealed that *Pax2* expression is down-regulated in *p53^−/−^* kidneys versus *p53^+/+^* (p = 0.037; n = 3 animals/group). This finding prompted us to test the hypothesis that *Pax2* is a p53 target gene. The details of the microarray will be published elsewhere (manuscript in preparation).

### *P53* and *Pax2* have overlapping expression in the developing kidney

**Figure 2:**
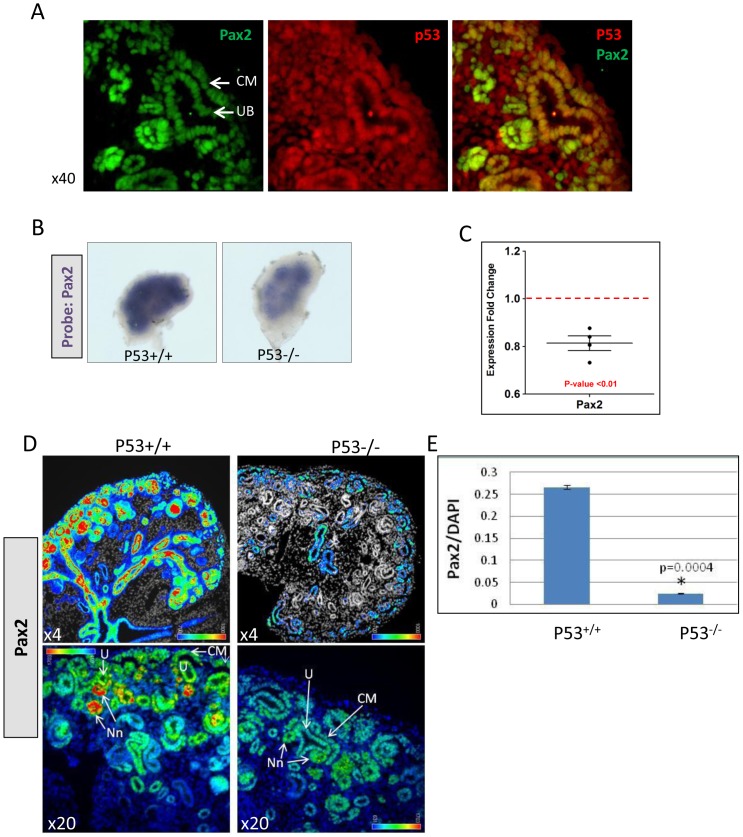
Decreased *Pax2* expression in *p53^−/−^* embryonic kidneys. **A**) p53 expression overlaps that of Pax2 in the developing kidney. Immunofluorescence staining was done on E15.5 kidney sections to visualize Pax2 and p53 protein expression. p53 co- localizes with Pax2 in the cap mesenchyme (CM) and in the ureteric tip (UB). **B**) In situ hybridization. Decreased expression of *Pax2* mRNA at E12.5. **C**) QPCR. *Pax2* mRNA is significantly lower in germ-line *p53^−/−^* kidneys. QPCR was done on RNA from individual E15.5 kidney pairs from wild-type and *p53^−/−^* embryos (n = 4). **D**) Pax2 protein in E15.5 kidneys, detected by IF staining. Both wild-type and mutant kidney sections were processed for IF staining simultaneously and images captured at identical exposure setting. After normalizing intensity to that in wild-type, images were converted to a heat-map to demonstrate differential Pax2 staining between wild-type and mutant kidneys. High intensity staining corresponds to red/yellow areas and lower intensity corresponds to green/blue. Top panels at x4 and bottom panels at x20. **E**) Quantitation of staining intensity was done using Slidebook software and is shown graphically. UB, ureteric bud/tips; CM, cap mesenchyme; Nn, nascent nephrons including pretubular aggregates and renal vesicles.

*p53* expression is developmentally regulated in the kidney [41]. At early stages (E11.5–12.5) *p53* expression is ubiquitous; however, it is progressively restricted to differentiating renal epithelia as postnatal development proceeds and declines to non-detectable levels in adult kidneys. *Pax2* expression is also developmentally regulated [11]. Pax2 is expressed throughout nephrogenesis in the *Six2*+ CM, in pretubular aggregates (PTA) and renal vesicles (RV) which are the differentiating nascent nephrons, in distal portions of the S-shaped body of the maturing nephron and in the ureteric tips/collecting ducts [12]. *p53* expression is more widespread in the mesenchyme than *Pax2*, showing expression in metanephric and stromal mesenchyme. Immunofluorescence staining demonstrated that p53 is expressed in the Pax2+ CM as well as in the UB (Fig. 2A).

### Decreased *Pax2* Expression in *p53^−/−^* Metanephroi

**Figure 3:**
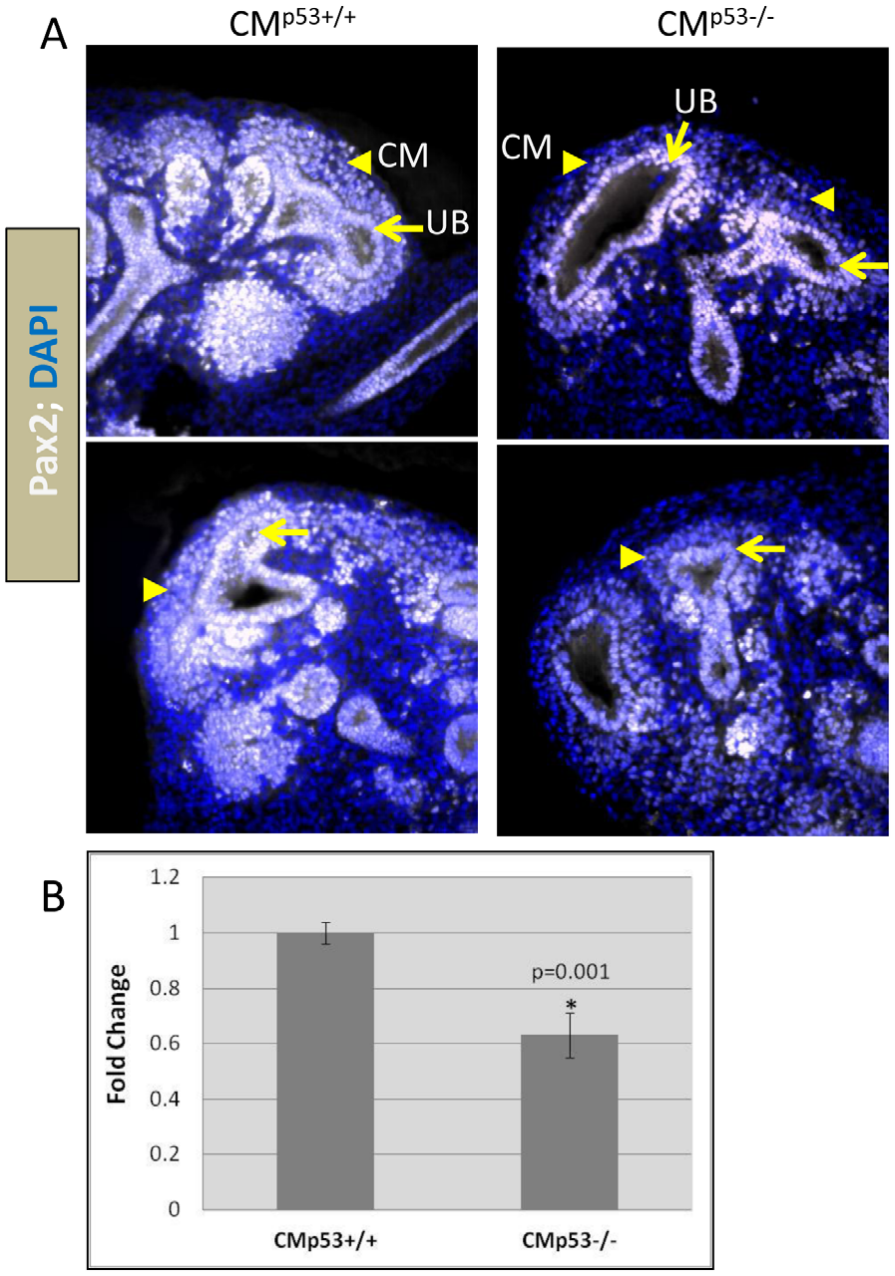
Decreased *Pax2* expression in cap mesenchyme of CM*^p53−/−^* kidneys. **A**) Reduced Pax2 staining in the CM of E12.5 CM*^p53−/−^* kidneys in comparison to wild-type CM, indicative of lower Pax2 protein levels. **B**) *Pax2* mRNA is ∼40% lower in CM*^p53−/−^* kidneys. QPCR was done on RNA from E15.5 littermate kidneys from wild-type (n = 5 pairs) and CM*^p53−/−^* (n = 5 pairs).

To validate our microarray findings, we examined *Pax2* gene expression in kidneys from *p53* ^−/−^ and *p53^+/+^* littermates. In situ hybridization revealed lower *Pax2* mRNA levels (Fig. 2B) in 4/11 (36%) null embryos, in line with the variable severity of renal hypoplasia in *p53^−/−^* mice [41]. To confirm the Pax2 ISH data, QPCR was done on RNA from individual E15.5 kidney pairs from wild-type (n = 4 pairs) and germ-line *p53^−/−^* embryos (n = 4 pairs). The scatter-plot in Fig. 2C shows a significant decrease in *Pax2* gene expression in individual mutant kidney pairs. In addition to decrease in *Pax2* transcript, immunofluorescence staining with Pax2 antibody of wild-type and mutant E15.5 kidney sections showed significantly decreased expression of Pax2 protein in both ureteric and mesenchyme lineages in *p53^−/−^* kidneys (Fig. 2D, E). Quantitation of staining intensity was done using Slidebook software and is shown graphically (Fig. 2E). Since kidneys with conditional *p53* deletion from CM cells recapitulate the nephron deficit phenotype of global *p53*-null kidneys, we examined *Pax2* expression in CM*^p53−/−^* kidneys. Intensity of Pax2 staining was markedly reduced in the cap mesenchyme of E12.5 CM*^p53−/−^* kidneys in comparison to wild-type CM (Fig. 3A) indicative of lower Pax2 protein levels. Moreover, *Pax2* mRNA is ∼40% lower in E15.5 CM*^p53−/−^* kidneys (Fig. 3B).

### ChIP-Seq Identification of p53 Bound to Multiple Regions in the *Pax2* Promoter in Developing Kidneys

**Figure 4:**
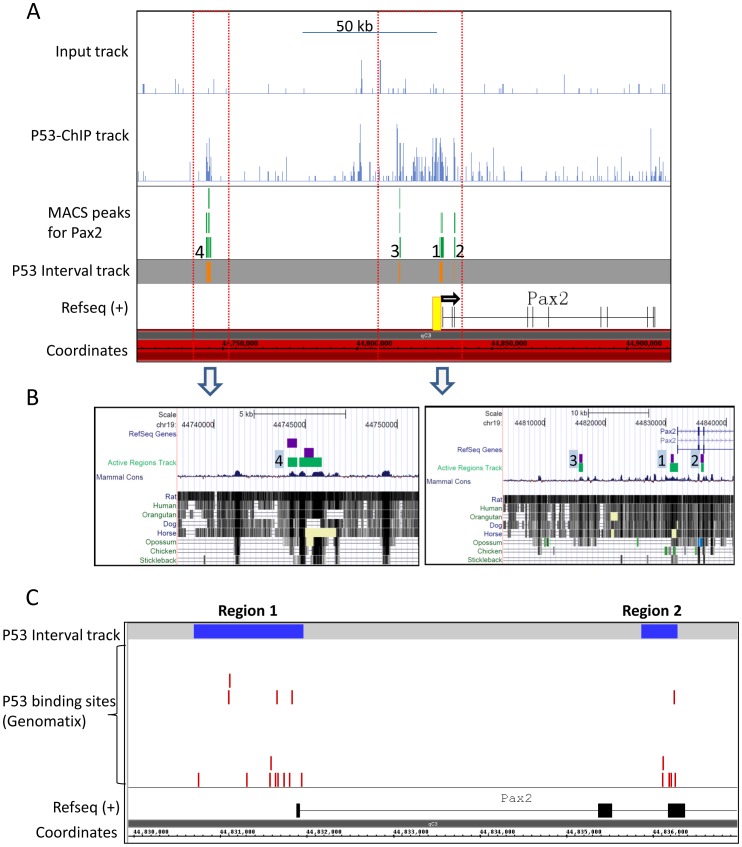
p53 occupancy at the *Pax2* gene locus in E15.5 kidneys identified by ChIP-Seq. A ) Genomic view of p53 occupancy at the *Pax2* gene locus identified by ChIP-Seq and visualized using the Integrated Genome Browser (IGB). Orange bars denote p53 enriched regions after background (Input) subtraction. Green bars show MACS peaks which represent points of highest density of sequenced fragments within the orange interval. Peak 1 and 2 are in the proximal promoter and in intron2-exon3, respectively. Two additional peaks were identified far distal to the *Pax2* gene at ∼−14 kb (peak 3) and ∼−83 kb (peak 4) from the transcription start site. Yellow box shows region validated for p53-enrichment by ChIP-PCR in Fig. 5. **B**) High sequence conservation in mammals of regions encompassing p53-enriched areas, including the non-genic distal regions 3 and 4, visualized in the UCSC genome browser (http://genome.ucsc.edu). **C**) Red bars show location of p53 binding motifs in and around p53-occupied regions 1 and 2 in the *Pax2* promoter/gene. Although p53 binding sites are broadly scattered across the entire region including the intervening region between regions 1 and 2 (see Fig. S3), p53 occupancy occurs at specific regions.

*In silico* analysis of the *Pax2* promoter revealed several putative consensus p53 binding sites, suggesting p53 may directly regulate *Pax2* expression during kidney development. To identify putative targets of p53 transcriptional function in the developing kidney, we performed ChIP-Seq using the Genome Analyzer (Illumina) on chromatin from E15.5 kidneys. The complete annotated data set of genes associated with p53-bound regions will be reported elsewhere. ChIP-PCR validation of several genes identified in the ChIP-Seq was recently published [48]. Figure 4 shows the ChIP tracks of p53 enrichment at the *Pax2* gene. Tracks for the Input (pre-ChIP) sample show non-specific background that was subtracted from p53-IP sample prior to peak-calling. p53 is enriched at the *Pax2* proximal promoter (Region 1) and at an intronic region (Region 2) (Fig. 4A). Region 1 extends from ∼−1200 bp to ∼+60 bp relative to the RefSeq annotated transcription start site (TSS) (http://www.ncbi.nlm.nih.gov/nuccore/NM_011037), with peak sequenced fragment density at −844 bp (Fig. 4A, green bars). p53 binding within the gene body encompasses a ∼400 bp region and overlaps intron2 and exon3, with peak fragment density in intron2. Two additional regions of high p53-occupancy were found at Regions 3 and 4 at −15 kb and −87 kb, respectively, from the *Pax2* TSS (Fig. 4A).

Multiple p53 binding motifs were identified in and around p53-occupied regions, thus validating specificity of the ChIP (Fig. 4C). Interestingly, while sequence conservation in mammals is high as expected for proximal promoter and gene sequences at Regions 1 and 2, respectively, non-genic distal p53-enriched regions 3 and 4 also show high sequence conservation (Fig. 4B). Non-genic conserved regions are postulated to function as transcriptional regulators [49]. Indeed, transcription factor binding site search (Genomatix) revealed p300 and CTCF binding sites, which are suggestive of enhancer function, flanking p53 binding sites in the −83 kb p53-enriched region (region 4). Therefore, we sub-cloned this ∼2 kb region upstream to SV40 minimal promoter in pGL3-promoter-luciferase reporter plasmid. However, reporter activity was not enhanced either at baseline or in response to p53 in metanephric mesenchyme mK4 cells (data not shown). Enhanced *Pax2* expression in response to p53 via this putative enhancer-fragment in other cell types (e.g., neural or intermediate mesoderm) cannot be ruled out. Collectively, these data strongly suggest that *Pax2* is a physiological target of p53-mediated transcriptional regulation.

### Differential p53 Binding at −844 in *Pax2* Promoter in mK4 (Pax2+) versus mK3 (Pax2-) Cells

**Figure 5:**
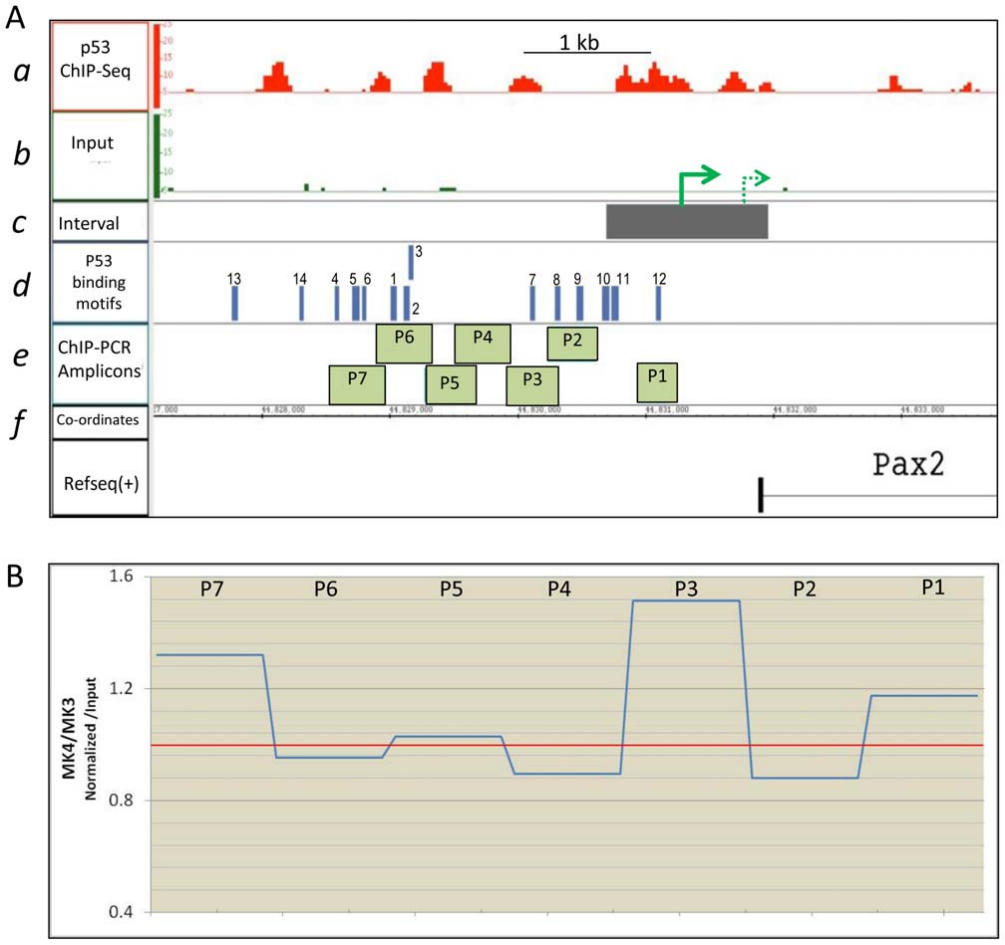
Differential p53 binding at −844 in *Pax2* promoter in mK4 (Pax2-expressing) versus mK3 (Pax2-non-expressing) cells. A ) (a) ChIP-Seq track showing p53 occupancy at *Pax2* proximal promoter (Region 1). (b) That this enrichment is not an artifact of amplification and sequencing is shown by lack of enrichment in the Input sample. Presence of any spurious false peaks in the Input sample eliminates that region from an interval, reflecting high stringency set for the MACS program. (c) Grey bar shows region of p53 enrichment designated as an interval. The dotted green arrow denotes RefSeq TSS, solid green arrow shows TSS described by [51]. (d) p53 motifs (blue bars) identified by Genomatix and manually. (e) Position of amplicons to validate ChIP-Seq data and show differential p53 occupancy between mK3 and mK4 cells by ChIP-PCR. (f) Co-ordinates on *mus* chromosome 19. **B**) Chromatin was immunoprecipitated from both cell lines with anti-p53 antibody and amplified by PCR. PCR band intensities were quantified using the Alphaimager software as described in Methods, and band intensity of immunoprecipitated fragments was normalized to Input band intensity for each cell line. Normalized values for each amplicon were plotted as mK4/mK3 ratios. Values greater than 1.0 indicate fragment enrichment in mK4 relative to mK3, as a result of increased p53 binding and immunoprecipitation as seen for amplicons P3 and P7 (blue line). Values are a mean of three independent ChIP experiments.

*Pax2* is expressed in induced metanephric mesenchyme derived mK4 cells, but not in mK3 cell line that represent early uninduced mesenchyme cells [50]. Both cell lines express p53 (Fig. S1). To determine whether p53 is differentially bound at the *Pax2* proximal promoter in Pax2-expressing versus non-expressing cells, chromatin was immunoprecipitated from both cell lines with anti-p53 antibody and amplified by PCR. Primers were designed to amplify regions shown to be p53 enriched in the immunoprecipitated sample over input by ChIP-Seq data (Fig. 5Aa, b). p53 motifs (blue bars in Fig. 5Ad) identified by Genomatix and manually are shown relative to TSS (Fig. 5Ac). Species-appropriate IgG was used as negative control. PCR band intensities were quantified using the Alphaimager software as described in Methods, and band intensity of immunoprecipitated fragments was normalized to Input band intensity for each cell line. Normalized values for each amplicon (Fig. 5Ae) were plotted as mK4/mK3 ratios (Fig. 5B). Values greater than 1.0 indicate fragment enrichment in mK4 relative to mK3, as a result of increased p53 binding and immunoprecipitation as seen for amplicons P3 and P7. Ratios for rest of the fragments showed values close to 1.0, indicating p53 binding in these regions is not enhanced in mK4 cells.

### p53 Activates the *Pax2* Promoter in Transient Transfection Assays

**Figure 6:**
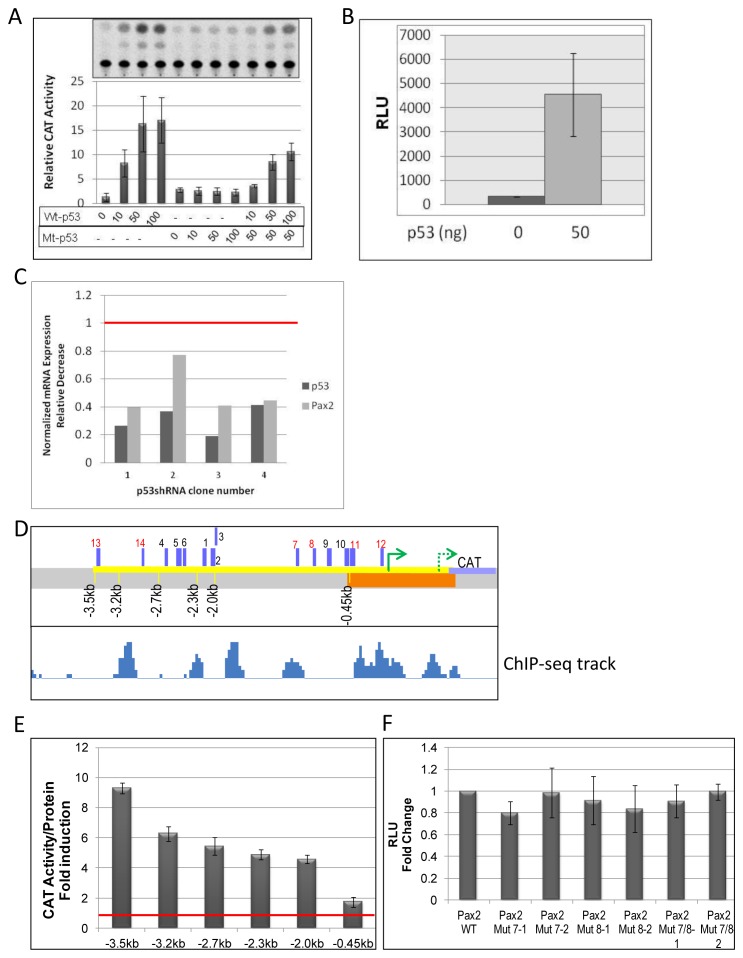
p53 trans-activates the *Pax2* promoter. **A**) *p53^−/−^* H1299 cells were co-transfected with a p53 expression plasmid (pCMV-p53) and a 4.1-Pax2-CAT reporter construct. Dose-dependent increase in reporter activity was observed with wild-type p53 (WT-p53). Reporter activity was maintained at baseline when reporter plasmid was co-transfected with a non-DNA binding mutant p53 (Mt-p53, pCMVp53-E258K). This mutant acts as a dominant-negative and inhibits *Pax2* promoter activation by wild-type p53. **B**) mK4 cells were transiently co-transfected with a p53 expression plasmid (pCMV-p53) and a 4.1-Pax2-Luc reporter construct. Absolute values of luciferase activity are plotted as Relative Light Units (RLU). Luciferase activity was normalized to protein concentration. **C**) p53 knock-down in mK4 cells using 4 different p53 shRNA plasmids showed corresponding decrease in *Pax2* mRNA, relative to mRNA levels from GFP- cells (dotted red line). mK4 cells were transfected with p53-shRNA-GFP plasmid, and GFP+ cells were sorted 48 h post-transfection, RNA harvested and used for QPCR. Gene expression was normalized to β-actin or GAPDH expression. **D**) Schematic of deletion mutant *Pax2* promoter constructs, shown with respect to p53 occupancy determined by ChIP-Seq. Orange bar denotes p53 occupancy in *Pax2* promoter, corresponding to region 1 in Fig. 2A. Green solid arrow shows TSS used in this study, dotted arrow denotes possible alternate TSS in RefSeq. Purple bars indicate location of p53 binding sites identified *in silico* and tested for p53 binding by EMSA, numbered 1–14. Mutagenized sites shown in red. **E**) Full-length or truncated Pax2-promoter-CAT reporter plasmids were transfected into UB cells either without or with pCMV-p53 (50 ng). CAT activity was normalized to protein concentration. Fold-induction by p53 of CAT activity over baseline (red-line) is plotted. **F**) Fold-change in reporter activity by p53 after site-directed mutagenesis of p53-binding site 7 (Mut 7–1 and 7–2) and 8 (Mut 8–1 and 8–2) individually or together (Mut 7/8). Two clones were tested in transient transfection assays per site mutated.

*In vivo* gene expression and ChIP data shown above strongly suggest that *Pax2* is a direct transcriptional target of p53. To further test this hypothesis, the 4.1-Pax2-reporter construct was co-transfected with a p53 expression plasmid into *p53^−/−^* H1299 human lung cancer cell-line. The 4.1-Pax2 BamHI/Not1 promoter fragment contains 3500 bp upstream to the transcription start site and ∼600 bp sequences in 5′UTR fused to the CAT or luciferase gene [51]. Greater than 15-fold increase in *Pax2* promoter activity was observed in this cell-line in the presence of wild-type p53 but not with a non-DNA-binding mutant of p53, p53-E258K (Mt-p53) (Fig. 6A), indicating the requirement for p53-DNA binding for transactivation to occur. Mt-p53 acts as a dominant-negative and inhibits transcriptional activity of wild-type p53. 4.1Pax2-reporter was also responsive to p53 in two kidney cell-lines tested, one from UB-lineage (see below) and another from mesenchyme (mK4). Kidney mesenchyme-derived mK4 cells represent induced cap mesenchyme cells [50]. Co-transfection of pCMV-p53 expression plasmid with 4.1Pax2-Luc resulted in greater than 20-fold increase in luciferase activity compared to baseline luciferase activity from transfectants that did not receive *p53* expression plasmid (Fig. 6B). Further, shRNA-mediated knockdown of *p53* in mK4 cells showed corresponding decrease in endogenous *Pax2* mRNA levels. *p53* knock-down was achieved by transfecting mK4 cells with 4 different p53 shRNA-GFP plasmids (Fig. 6C). GFP ‘+’ and ‘-‘ cells were separated 48 h after transfection by FACS. *P53* and *Pax2* mRNA levels from GFP+ cells were normalized to levels from GFP- cells. Transfection with scrambled control shRNA plasmid did not result in decreased *p53* or *Pax2* mRNA levels (not shown).

Collectively, our results thus far show that not only is the *Pax2* promoter highly responsive to p53 but is also p53-bound both in kidneys and mK4 cells. The p53 binding motifs identified by Genomatix along the *Pax2* promoter were located within or in close proximity to identified p53 peaks identified by ChIP-Seq (Fig. 6D, numbered 1–14). To test the functional relevance of the identified sites, serial truncations were made to the full-length promoter 4.1-Pax2 which has 3500 bp of DNA upstream of the TSS (Fig. 6D and E). Although the deletion constructs show increased CAT activity in response to p53, relative activity of the truncated constructs was consistently lower than from the full-length construct (Fig. 6E). The deletion constructs −3.2kbPax2-CAT and −0.45kbPax2-CAT exhibited 50% and 80% reduction in reporter activity in response to p53, respectively.

The p53 binding motifs along the *Pax2* promoter (Fig. 6D, numbered 1–14) were tested for p53 binding. In EMSA, recombinant p53 showed weak binding to multiple sites (Fig. S2A). A consensus, high affinity p53 binding site [52] was used as positive control. The consensus sequence showed robust binding to p53 (Fig. S2A, complex 1). *Pax2* promoter sites, however, showed weak binding. Further, several sites generated a higher mobility p53-DNA complex (complex 2), possibly from less multimerized p53 at these sites; this complex was not observed with the consensus site. To circumvent labeling efficiency issues for any probes, competition EMSA was done using p53 consensus sequence as probe and competing its binding to recombinant p53 with the identified putative sites. Binding of the p53 consensus oligoduplex to purified p53 was abolished or attenuated by addition of 200-fold excess of unlabeled p53 consensus sequence (self) or by the p53 binding site in the *p21* gene (Fig. S2B, lanes 2–3). However, p53 binding sites in the *Pax2* promoter competed weakly or not at all for p53 binding. Competition obtained with different sites was quantified (Fig. S2C).

To identify a specific site responsible for *Pax2* transactivation, site-directed mutagenesis was done on the full-length promoter construct. Not all peaks contain consensus binding site sequences. We picked the following sites for site-directed mutagenesis because – sites 7 and 8 showed p53-binding by EMSA, sites 11 and 12 occur within the interval and peak region, and sites 12 and 14 showed high percent conservation between mammals (Table 1). Percent conservation is the percentage of nucleotides predicted to be conserved in 30 mammalian species, calculated using the UCSC browser built-in method (Placental Mammal Basewise Conservation by PhyloP) (Methods S1). As the −3.2 kb deletion construct (without site 13) showed decreased CAT activity relative to the −3.5 kb construct (with site 13) (Fig. 6E), we also tested site 13 for functionality and response to p53 *in vitro*. Sites mutated by site-directed mutagenesis were re-tested by EMSA to confirm lack of binding to p53 (not shown). Point mutagenesis of single or double sites did not decrease reporter gene activity in response to p53 (Fig. 6F, and data not shown). Since multiple weak sites may promote p53 binding at the *Pax2* promoter and transactivation co-operatively, further individual site mutagenesis was not pursued. In summary, our data from ChIP, p53 over-expression and promoter deletion analysis indicate that p53 binds to and transcriptionally activates the *Pax2* promoter.

**Table 1:** Percent conservation in mammals of p53 binding sites in the *Pax2* promoter.

Binding site	Location on Chromosome 19	Percent Conservation	Oligonucleotide sequence 5′→3′
1	44829010–44829059	43.75%	cagacatacc aagacttaac ttaagcacac ccaacaagag ttcctttatg
2	44829110–44829159	68.00%	aaggaagata aggctactgc ggttactacc accttagagc ttctggctcc
3	44829150–44829189	72.50%	ttctggctcc ccagtatggg agctgaccct tttaggctga
4	44828571–44828608	63.16%	ttg attatctaga cccagaaacc agctggaaaa ctaac
5	44828707–44828769	42.86%	cct gggaaggcat gcctgagttt tctggagcta catagaaatt agactatcac tagccctcat
6	44828785–44828821	40.54%	tgtgg cagatacttg cgtaagtgca acttcaaaag tg
7	44830101–44830143	72.09%	ggga gcgagtgcgg gccaggctgc agagactggg ccagcggct
8	44830295–44830340	74.42%	gcatcctctg tcactgccgc ggacaagccg ctgcgcaccg tctccc
9	44830465–44830518	59.57%	aggcgtctgg actgcccaga ctccggggag ggggtgcgcg ccaccgcgtg tggg
10	44830665–44830724	60.00%	gaactcagcg caagggtagc cgagttagga caggggctgc ggactcgcgg gcgtctggga
11	44830735–44830794	73.33%	ttggccggtg gatggcaggg ctgggcgagt taggactgag agcctggctt cggagtgccg
12	44831085–44831124	97.50%	gaatctattg cctttgtctg acaagtcatc catctcccgg
13	44827769–44827817	40.82%	ggtta gcagacagga tctctgcctg cactttactt atgcatgatc cgcc
14	44828298–44828333	94.44%	aaccaa gcctgacagg tcgagccctg gctgtgtttg

### Epistasis between *p53* and *Pax2*

**Figure 7:**
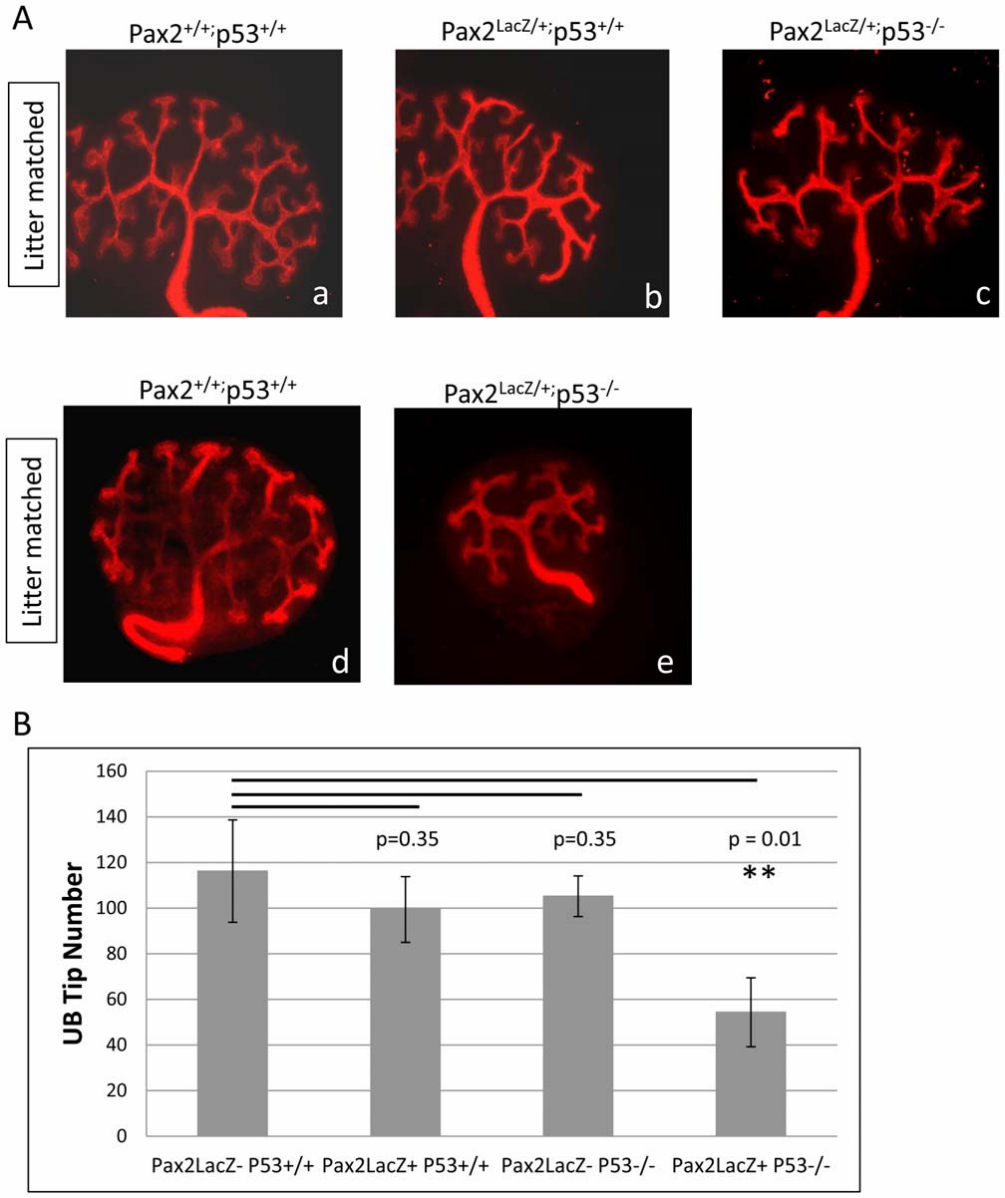
Epistasis between *p53* and *Pax2*. **A**) E13.5 kidneys from *Pax2^LacZ/+;^p53^+/−^* crosses were stained with cytokeratin and UB tips counted. **B**) UB tip number was used as a phenotypic readout and measure of renal hypoplasia in *Pax2^LacZ/+;^p53^−/−^* animals compared to wild type, or *Pax2^LacZ/+^;p53^+/+^*. Reduction of either *Pax2* gene dosage or elimination of *p53* gene reduced UB tip number by ∼12–15%. Complete *p53* deficiency with *Pax2* haploinsufficiency resulted in ∼55% decrease in tip number. For each genotype, N = 3−5 kidney pairs from at least 3 independent litters. All animals were harvested at ∼E13.0, cultured overnight and fixed for whole mount cytokeratin staining to enable counting of UB tips.

In addition to regulating each other ([53] and this study), both Pax2 and p53 independently regulate a cohort of genes. To determine whether an epistatic relationship exists between the two genes, we reduced *p53* gene dosage on a *Pax2^+/−^* background by crossing *p53^+/−^* to *Pax2^LacZ/+^* mice which contain the *β-galactosidase* gene knocked-in to the *Pax2* locus. *Pax2* haploinsufficiency is known to result in reduced kidney size with lower UB tip and nephron number [16]. Similarly, *p53^−/−^* kidneys exhibit renal hypoplasia with fewer UB tips [41]. Thus, we used UB tip number as a phenotypic readout of renal hypoplasia in *Pax2^LacZ/+;^p53^−/−^* animals. On this mixed genetic background (*C3H/He; C57BL6*), reduction of either *Pax2* gene dosage or elimination of *p53* gene reduced UB tip number by ∼12–15% (Fig. 7A, B). However, complete *p53* deficiency superimposed on *Pax2* haploinsufficiency resulted in ∼55% decrease in tip number, suggesting functional co-operativity between the two transcription factors.

## Discussion

In this study we have shown that *Pax2* is a physiological target for p53-mediated transcription in the embryonic kidney. *Pax* family of transcription factors plays a key role in lineage specification and organogenesis. *Pax2* expression is essential for development of the urogenital system and mid-hindbrain patterning [15], [54]. While complete loss of *Pax2* expression results in renal agenesis, *Pax2^+/−^* kidneys are hypoplastic. *Pax2* loss in the CNS results in exencephaly [55]. *p53* expression is ubiquitous early in embryogenesis. P53 activity is essential for mesodermal determination in co-operation with Smads [40], [56], [57]. *Pax2* and *p53* expression overlaps temporally and spatially in metanephric development, when expression is ubiquitous in the ureteric epithelia and surrounding metanephric mesenchyme. *p53* is expressed in all cells of the embryo up to E10.5 [31], thus its expression precedes that of *Pax2* in the intermediate mesoderm. However, *Pax2/8* expression is required to induce and specify the nephric lineage [11]. Unlike *Pax2^−/−^* mutants, renal agenesis has never been observed or reported in *p53^−/−^* embryos suggesting a modulatory role for p53 in regulating *Pax2* expression rather than an on-off regulation. Indeed, this is in line with the findings of this study, since elimination of p53 activity results in decreased *Pax2* expression but not its complete loss in *p53^−/−^* kidneys.

The 4.1Pax2 promoter-reporter construct, which contains the kidney- and mid-hindbrain-specific enhancers faithfully recapitulates endogenous *Pax2* expression pattern in the intermediate mesoderm and neural tissue *in vivo*. The transgene is expressed in the nephric duct epithelia and its derivatives but not in the metanephric mesenchyme [51]. The ability of this promoter fragment to exhibit relatively high baseline activity and response to p53 in mesenchyme mK4 cells reflect inherent differences between *in vivo* and *in vitro* models. However, decreased *Pax2* expression with *p53*-knockdown in these cells indicates p53 does positively regulate *Pax2* expression in both mesenchyme and UB cells.

Our data does not rule out the possibility of an intermediate factor X as regulator of *Pax2* promoter activity. However, p53 is enriched at the *Pax2* promoter in mK4 cells that express *Pax2* but not in *Pax2* non-expressing mK3 cells, although *p53* is expressed in both cell lines. This finding, along with collective *in vivo* data showing decreased *Pax2* expression in germ-line *p53^−/−^* and conditional CM*^p53−/−^* kidneys, along with *in vitro* transient transfection data strongly suggest *p53* is directly upstream to *Pax2*. Unlike the deletion mutants, none of the point-mutants showed decreased activation by p53 (Fig. 6F and data not shown). Since DNA-binding activity of p53 is required to activate the *Pax2* promoter (Fig. 6A) we surmise that multiple binding sites may function co-operatively to enrich p53 at the *Pax2* promoter and enhance transcription.

p53 chromatin occupancy profile in mK4 cells overlaps with the *in vivo* occupancy profile at the *Pax2* proximal promoter region, although the resolution offered by ChIP-Seq is far greater than by ChIP-PCR. Also, whole embryonic kidneys were used for chromatin preparation for ChIP-Seq. Thus, the relative differences in p53 occupancy between mK4 cells and kidneys probably reflect differential binding in different cell types.

**Figure 8:**
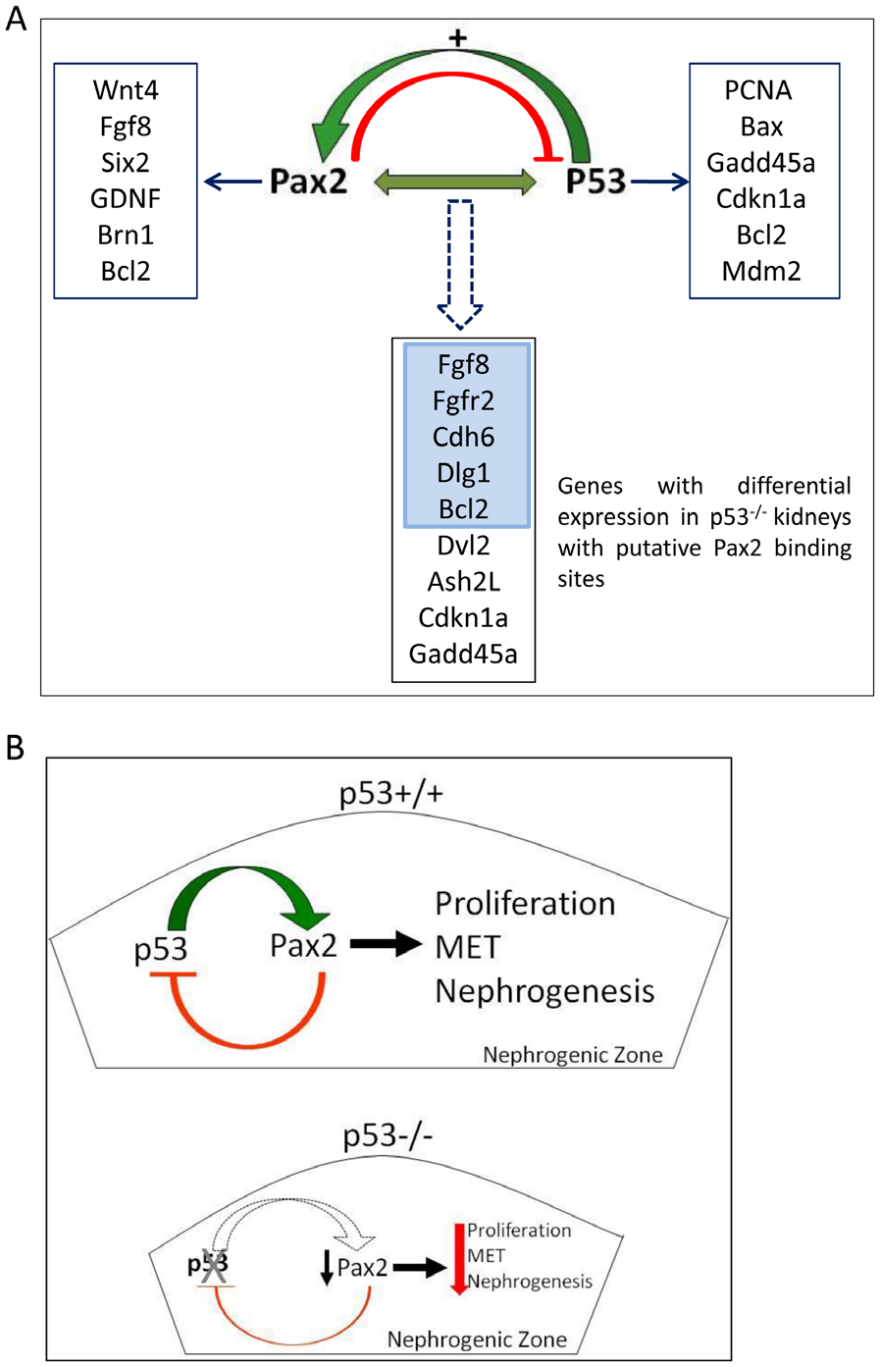
**A) Model for autonomous and interactive gene regulation by p53 and Pax2.** Genes regulated independently by each transcription factor. P53 positively regulates Pax2 expression, and the two may co-operatively regulate genes shown in box below dotted arrow. Expression of these genes is altered in *p53^−/−^* E15.5 kidneys and these genes contain p53-binding motifs that are occupied by p53 in vivo as determined by ChIP-Seq. Genomatix search revealed Pax2 binding motifs in these genes. **B)** **Model for p53-Pax2 cross-talk in the developing kidney.** p53 enhances *Pax2* expression in mesenchyme cells and promotes their transition to epithelia. Thus, p53 serves to promote differentiation of progenitor cells to nascent nephrons, and in its absence Pax2 down-regulation is a possible mechanism of nephron deficit observed in these kidneys.

The synergistic decrease in tip number implies that in addition to autonomously regulating gene expression in the kidney, p53 and Pax2 may co-regulate genes and demonstrate epistasis to each other (Fig. 8A). We identified Pax2 binding sites in a subset of genes with altered expression in *p53^−/−^* kidneys and that are bound by p53. Protein products of these genes (*Fgf8, Fgfr2, Dlg1, Cdh6*, and *Bcl2*– Fig. 8A, blue box) have been shown to be required for kidney development [58]–[64]. While both transcription factors regulate distinct repertoire of genes in response to specific stimuli independent of each other [20], [65]–[69], we propose some known p53 (*Gadd45a, Cdkn1a*) [65], [70] or Pax2 (*Fgf8*) [69] target genes may be regulated co-operatively by p53 and Pax2 in the kidney. The validity of this model remains to be tested.

**Table 2:** ChIP-PCR primer sequences.

Primer	Chr.19: Amplicon Start	Chr.19: Amplicon End	Amplicon (bp)	Sequence
P1	44830940	44831180	240	F atcctcaccctccctcttc
				R tgtgtctctctaaaagctgc
P2	44830233	44830617	384	F ttcatagcctcctcctctcc
				R gtccttccctttcgcctt
P3	44829920	44830314	394	F gcctgggaggagggagcgaa
				R cggcagtgacagaggatgc
P4	44829512	44829938	426	F gtttcttactttccagcag
				R ttcgctccctcctcccaggc
P5	44829275	44829678	403	F ttccctatgagaagttagtg
				R acattctgggtgagtttcagg
P6	44828895	44829328	433	F tatccattgtctatgttcaca
				R caaatctcacaacctcaca
P7	44828544	44828972	428	F cttccaaagcagcagggtag
				R gttcctcactccaacagagat

The ability of both p53 and Pax2 to associate with multiple transcriptional regulatory complexes, as well as their ability to recruit chromatin modifying enzymes to regulate expression of genes encoding transcription factors they physically associate with, would allow p53-Pax2 cross-talk to integrate various stimuli in different developing tissues. Depending on the interacting partners, both transcription factors can activate or repress transcription. Our data places *p53* upstream to *Pax2* in the developing kidney. The increased severity of hypoplasia in *Pax2^LacZ/+^;p53^−/−^* double mutants relative to single mutants suggests interactions beyond a linear genetic pathway. The two proteins may either directly or as part of a larger complex, or combinatorially, bind to and co-regulate transcription of genes. For example, several putative p53 target genes (altered expression in *p53^−/−^* kidneys and show p53 occupancy *in vivo -* our unpublished data) that also contain Pax2 binding sites (Fig. 8A, below dotted arrow) may be co-regulated.

Pax2 positively regulates expression of pro-survival gene *Bcl2* [71] whereas p53 is a known transactivator of pro-apoptotic genes such as *Bax*, *PUMA* and *Noxa* [67], [72], [73]. *Pax2* deficiency results in increased apoptosis [17], [74]. Although this study did not compare the apoptotic index between wild-type, single and *Pax2^LacZ/+^;p53^−/−^* double mutants, the worsening hypoplasia upon *p53* deletion in *Pax2^LacZ/+^* kidneys suggests increased apoptosis may result from further *Pax2* decrease as a result of *p53* loss. Effects of *p53* deletion are genetic background dependent. *Pax2^LacZ/+^* mice are on a *C3H/He* background [13]. When *p53^−/−^* mice are crossed to this background, these mixed background mice did not show as severe a renal phenotype as we observed in *C57BL6* mice.

Given that *Pax2* is a proto-oncogene, promotes cell survival and is up-regulated in Wilm’s tumour and renal cell carcinoma [10], [75], why does p53, a tumor suppressor, positively regulate *Pax2* expression? In addition to lineage specification and promoting cell survival, *Pax2* is also implicated in promoting mesenchyme-to-epithelial transition and cell differentiation [76]. We propose a feed-back model wherein Pax2 in the nephrogenic zone down-regulates *p53* expression [53] in the self-renewing cap mesenchyme (Fig. 8B). Our data from conditional *p53*-deletion kidneys (CM*^p53−/−^*) show recapitulation of renal hypoplasia observed in germ-line *p53^−/−^* kidneys. CM*^p53−/−^* kidneys exhibit decreased Pax2 levels specifically in the CM (Fig. 3A) indicating that p53 levels in the CM are sufficient to enhance *Pax2* expression in mesenchyme cells and promote their transition to epithelia. An alternate explanation for decreased Pax2 staining in the CM may be precocious differentiation of CM to nascent nephrons. To address this possibility, we stained embryonic and post-natal kidneys sections with Lhx1, a marker for nascent nephrons. Not only is Lhx1 staining decreased in the mutant kidneys (Fig. 1D), but these kidneys showed no ectopic Lhx1+ differentiated structures that would be expected if these cells were undergoing precocious differentiation. Modulation of p53 function by Pax2 may be viewed as a cell survival mechanism, since unconstrained p53 activity results in excessive apoptosis [46].

We conclude that p53 serves to promote differentiation of progenitor cells to nascent nephrons, and in its absence *Pax2* down-regulation is a possible mechanism of renal hypoplasia observed in these kidneys. We propose that the cross-talk between p53 and Pax2 provides a transcriptional platform that promotes nephrogenesis, thus contributing to nephron endowment. Our data contribute to further elucidate mechanisms regulating *de novo* nephrogenesis in the embryonic kidney, an area of interest not only for potential use in *in utero* intervention but also from the point of regenerative medicine.

## Materials and Methods

### Mice

*p53^+/−^* mice on *C57BL/6* background (Jackson labs) were time-paired to obtain E15.5 *p53^+/+^* and *p53^−/−^* litter-matched kidneys. For conditional *p53* deletion in cap mesenchyme, *Six2^GC^;p53^loxP/+^* mice were crossed to *p53^loxP/loxP^* mice and embryonic kidneys obtained from resultant crosses. The *Six2^GC^* mice were a gift from A. McMahon [3]. *Pax2^LacZ/+^* knock-in mice were a kind gift of M. Bouchard, and maintained on a *C3H/He* background [13]. For epistasis experiments, *Pax2^LacZ/+;^p53^+/−^* mice were generated and crossed to *p53^+/−^* to obtain *p53^−/−^* mice on a *Pax2^+/−^* background. All animal protocols utilized in this study were approved by and in strict adherence to guidelines established by the Tulane University Institutional Animal Care and Use Committee.

### Immunofluorescence Staining

E15.5 kidneys were fixed in 10% formalin and processed for paraffin embedding and sectioning. Five micrometer paraffin sections were subjected to antigen retrieval (10 mM sodium citrate, pH 6.0) after deparaffinization and rehydration. Immuno-staining was done as previously described [47]. Antibodies for the following proteins were used: p53FL (SantaCruz, 1∶500) and Pax2 (1∶200, Zymed/Invitrogen). DAPI (1∶500, D1306, Invitrogen) was used to stain nuclei. Whole-mount staining was done on E12.5–13.5 kidneys as previously described [41] with Pan-cytokeratin antibody (Sigma, 1∶200). The immunofluorescent images were captured using a 3D or deconvolution scope (Leica DMRXA2) and staining intensity was quantified using Intelligent Imaging Innovations SlideBook software.

### Chromatin Immunoprecipitation (ChIP) Assays

Chromatin was prepared from 15 pairs of E15.5 kidneys. After shearing, p53-bound chromatin was immunoprecipitated with p53FL antibody (SantaCruz) and subjected to ChIP coupled to next generation sequencing (ChIP-Seq) using the Illumina protocol on the Solexa GAII. p53 peaks were called using the MACS program [77]. P53 antibody (Santa Cruz, SC-6243x) was used for all p53 ChIP experiments. ChIP-PCR on mK3 and mK4 cells was performed using reagents and protocols from the Upstate Biotechnology ChIP kit with modifications as previously described [78]. PCR primer sequences are shown in Table 2. ChIP tracks showing p53 occupancy at the *Pax2* gene locus were generated by Integrated Genome Browser (IGB; http://bioviz.org/igb) using ChIP-Seq-derived sequence files.

### *In situ* hybridization

Whole mount in situ hybridization was performed as described [79]. E10.5-E11.5 mouse embryos or E12.5 kidneys were fixed overnight in 4% formaldehyde in PBS, then dehydrated in methanol and stored at −20°C until use. Digoxigenin-dUTP labeled RNA probes were used at 0.5 µg/ml. Alkaline phosphatase-conjugated anti-digoxigenin Fab fragments were used at 1∶5000. Color reactions were carried out overnight. Embryonic tissue was photographed in glycerol.

### p53 knockdown and RT-qPCR

mK4 cells were transfected with p53-shRNA-GFP plasmid (SuperArray Biosciences, KM0931G) for 72 h. Transfected cells were sorted by FACS and RNA was isolated from GFP^+^ and GFP^-^ cells. Real-time primers for *p53* (TaqMan Gene Expression Assay, ID Mm01731287_m1) and *Pax2* (TaqMan Gene Expression Assay, Mm01217939_m1) were from Applied Biosystems. The TaqMan expression assay was constituted using reagents from the Brilliant II QRT–PCR 1-step Master Mix kit (Agilent Technologies). The thermal profile used was as follows: 50°C for 30 min, 95°C for 10 min and 45 cycles of 95°C for 15 s, 56°C for 1 min and 72°C for 30 s. The reactions were done in triplicate. The scale bars represent the standard error of mean. Expression was normalized to *GAPDH* or *β-actin* levels.

### Plasmids

4.1Pax2-Luc, 4.3Pax2-CAT and truncated Pax2-CAT constructs were a kind gift of G. Dressler [51]. The *p53* expression vectors have been described previously [80]. Site-directed mutagenesis was done using the QuickChangeTM Site-directed Mutagenesis System (Stratagene, La Jolla, CA) as per manufacturer’s directions.

### Cell Culture, Transient Transfection, and Reporter Assays

*p53*-null human lung carcinoma cells H1299 (ATCC), an E11.5 mouse ureteric bud cell line (UB cells; kind gift of J. Barasch) and mouse kidney cap mesenchyme cell lines (mK3 and mK4 cells; kind gift of S. Potter) were maintained in media supplemented with 10% fetal bovine serum, penicillin (100 units/ml), and streptomycin (100 µg/ml) at 37°C in a humidified incubator with 5% CO_2_. Cells were transfected with 1.0 µg of DNA/well promoter-reporter vectors along with pCMV-p53-(wt) or pCMV-p53-(mutant) expression plasmids (0–250 ng). Transfection was performed using the Lipofectamine Plus reagent (Invitrogen) according to the manufacturer’s recommendations. Four hours after transfection, fresh medium was replaced, and cell extracts were prepared 24–48 h later using a reporter lysis reagent (Promega). Aliquots of cell lysate were analyzed for CAT or luciferase activity after normalization for protein content or β-galactosidase activity as previously described [81].

## Supporting Information

Figure S1 **A) p53 is expressed in both mK3 and mK4 cells. Western blot was done on whole cell lysates, and B) Pax2 is expressed in mK4 cells but not in mK3 cells.**
(TIF)

Figure S2 **EMSA shows p53 binding to** ***in silico*** **identified sites in and around the p53-enriched region in the** ***Pax2*** **promoter.** A) ^32^P-labelled oligonucleotides (Table 1) were incubated with recombinant, purified C terminus-truncated and constitutively active p53. C, p53 consensus sequence ( [52]). Free probe and DNA-p53 complexes are indicated. B) Competition by p53 binding sites identified in Pax2 promoter with the p53 consensus sequence. Labeled consensus oligoduplex was incubated with p53 alone (lane 1) or in presence of unlabelled competitor oligoduplexes as described in Methods S1. Diminished consensus-p53 complex indicates effective competition. C) Inhibition of complex formation between p53 and consensus binding site by addition of various unlabelled competitor oligoduplexes was quantified and plotted. Unlabelled consensus binding site and p21 promoter-p53 binding site compete effectively for p53 binding, whereas p53 binding sites from the Pax2 gene region (sites 1–14) show weak competition.
(TIF)

Figure S3 **p53 binding sites are broadly scattered across the entire region including the intervening region between regions 1 and 2.** IGB view of Chromosome 19∶44,830,572 - 44,836,404 is shown. a) Location of p53 binding motifs identified by Genomatix; b) Vertical green bars show p53 binding sites in p53-occupied region denoted by horizontal green bar; c) p53 ChIP-track; d) Input track; e) *Mus* Chromosome 19 coordinates.
(TIF)

Methods S1 **Supporting methods file.**
(DOCX)
