# Supplementary figures and images for: A p53-Pax2 Pathway in Kidney Development: Implications for Nephrogenesis
Source: PLoS One. 2012 Sep 12;7(9):e44869. doi: 10.1371/journal.pone.0044869 (PMC3440354; doi:10.1371/journal.pone.0044869)

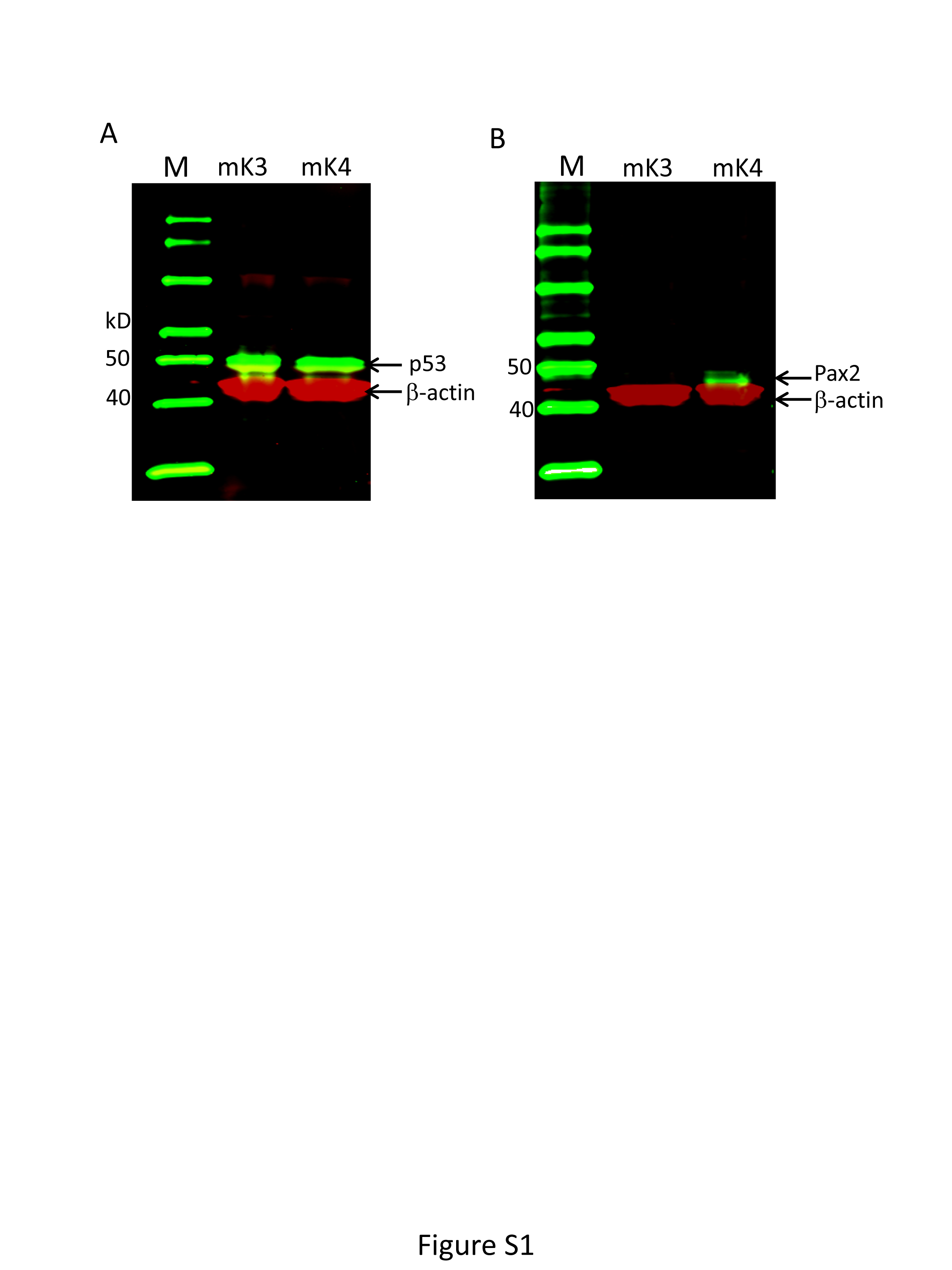

Supplement: Figure S1 — A) p53 is expressed in both mK3 and mK4 cells. Western blot was done on whole cell lysates, and B) Pax2 is expressed in mK4 cells but not in mK3 cells. (TIF) [file pone.0044869.s001.tif]

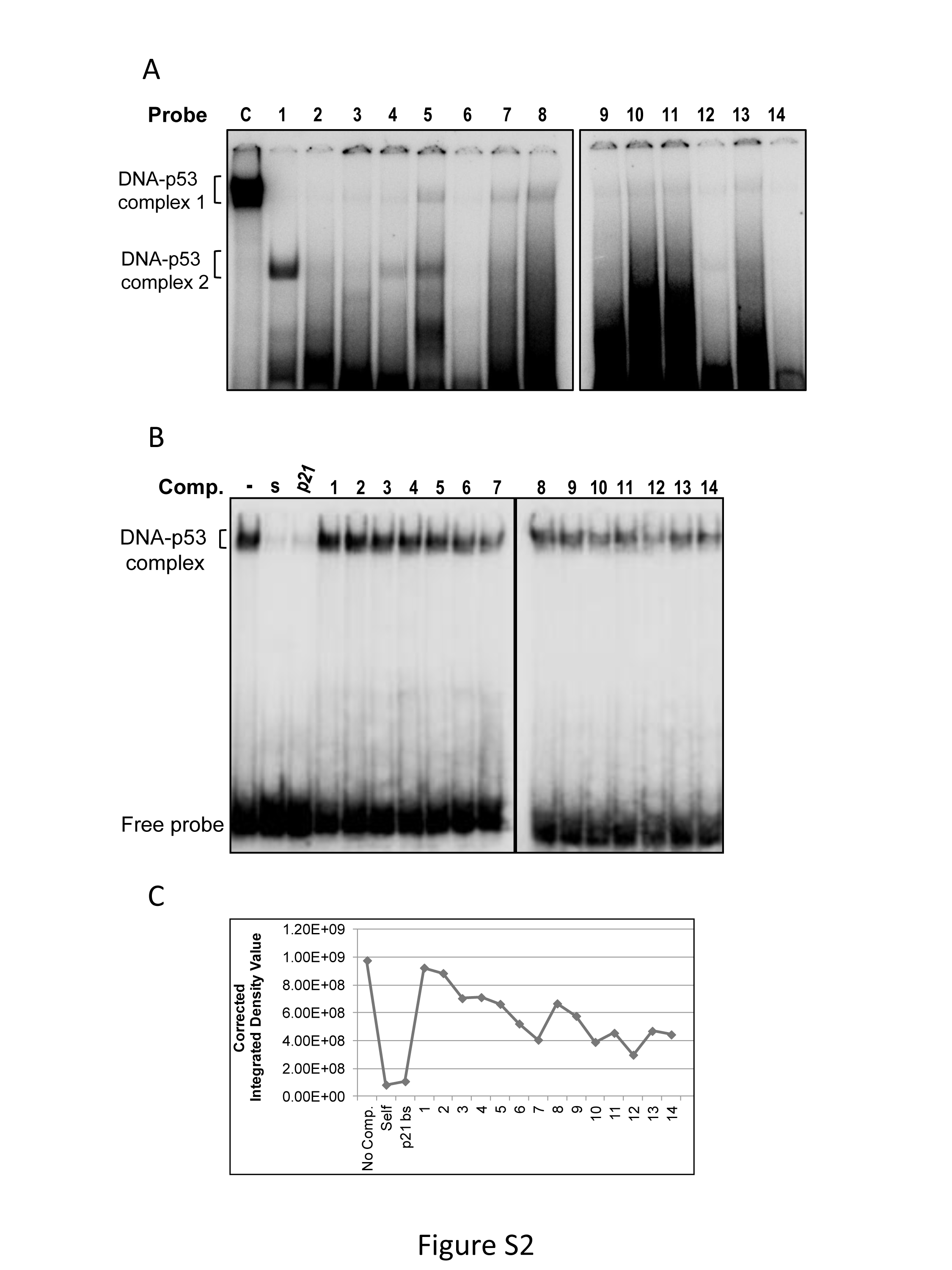

Supplement: Figure S2 — EMSA shows p53 binding to in silico identified sites in and around the p53-enriched region in the Pax2 promoter. A) 32P-labelled oligonucleotides (Table 1) were incubated with recombinant, purified C terminus-truncated and constitutively active p53. C, p53 consensus sequence ( [52]). Free probe and DNA-p53 complexes are indicated. B) Competition by p53 binding sites identified in Pax2 promoter with the p53 consensus sequence. Labeled consensus oligoduplex was incubated with p53 alone (lane 1) or in presence of unlabelled competitor oligoduplexes as described in Methods S1. Diminished consensus-p53 complex indicates effective competition. C) Inhibition of complex formation between p53 and consensus binding site by addition of various unlabelled competitor oligoduplexes was quantified and plotted. Unlabelled consensus binding site and p21 promoter-p53 binding site compete effectively for p53 binding, whereas p53 binding sites from the Pax2 gene region (sites 1–14) show weak competition. (TIF) [file pone.0044869.s002.tif]

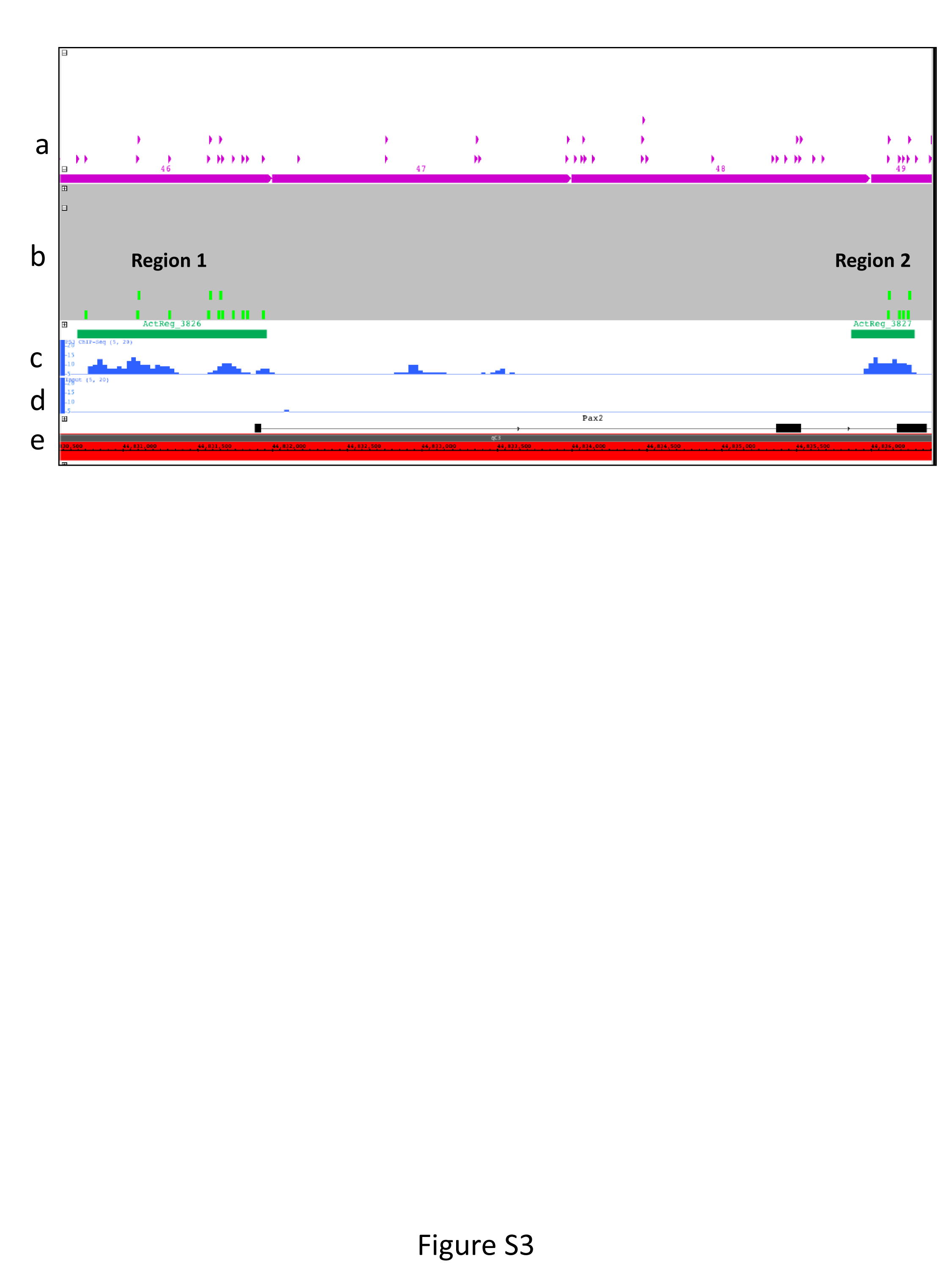

Supplement: Figure S3 — p53 binding sites are broadly scattered across the entire region including the intervening region between regions 1 and 2. IGB view of Chromosome 19∶44,830,572 - 44,836,404 is shown. a) Location of p53 binding motifs identified by Genomatix; b) Vertical green bars show p53 binding sites in p53-occupied region denoted by horizontal green bar; c) p53 ChIP-track; d) Input track; e) Mus Chromosome 19 coordinates. (TIF) [file pone.0044869.s003.tif]
