# Supplementary material for: A p53-Pax2 Pathway in Kidney Development: Implications for Nephrogenesis
Source: PLoS One. 2012 Sep 12;7(9):e44869. doi: 10.1371/journal.pone.0044869 (PMC3440354; doi:10.1371/journal.pone.0044869)
Supplement: Methods S1 — Supporting methods file. (DOCX) [file pone.0044869.s004.docx]

**SUPPLEMENTAL METHODS**

**Electrophoretic Mobility Shift Assay (EMSA)** *—* Double-stranded oligonucleotides were end labeled with [^32^P]dATP with T4 kinase for use as probes in the gel mobility shift assays as previously described [[81](#_ENREF_81)]. The labeled probe was incubated for 20 min at room temperature with 0.5µg recombinant, purified C-terminus truncated, constitutively active p53, a kind gift from Dr. Michelle Barton at MD Anderson Cancer Center, Houston. Specific competitor oligonucleotides were added in 200-fold molar excess 15 min before addition of the radioactive probe. 25,000cpm labeled probe was used for each reaction. P53-probe complexes were quantified using the AlphaImager software and plotted as Integrated Density Value after background subtraction (Fig. S2C).

**Calculation of percent conservation for EMSA sites** - The p53 binding motifs that were localized in UCSC browser were analyzed for the Placental Mammal Basewise Conservation by PhyloP. In this analysis, the program statistically calculated the genomic conservation of those motifs from 30 vertebrate species; the sites of nucleotides predicted to be conserved are assigned positive scores, while sites predicted to be fast-evolving are assigned negative scores, for each of which the relative frequency was calculated. The cumulative relative frequency (CRF) interprets the percentage of all the fast-evolving sites; on the other hand, “1.0-CRF” interprets the percentage of all the conserved sites, defined as the percentage conservation.

**Microarray –** RNA was prepared from three pairs of litter-matched *p53^+/+^* and *p53^-/-^* E15.5 kidneys, amplified, labeled and the cDNA hybridized to Agilent 4x44K Whole Mouse Genome Microarray slide from Agilent Technologies. To prevent bias from signaling detection on different colors (Cy5 – Red, Cy3 – Green), we applied dye-swap strategy to this experiment, in which identical pairs of *p53^+/+^* & *p53^-/-^* cDNA samples were reversely labeled by Cy5 and Cy3 into two groups. Raw data were analyzed using Gene Spring software (<http://www.genomics.agilent.com>). Only genes, showing a significant (p<0.05) differential change in expression after Benjamini and Hochberg false discovery rate correction in the three datasets were used for further analyses.

**Supporting Information Legends**

**Fig. S1**. **A)** p53 is expressed in both mK3 and mK4 cells. Western blot was done on whole cell lysates. **B)** Pax2 is expressed in mK4 cells but not in mK3 cells.

**Fig. S2. EMSA shows p53 binding to *in silico* identified sites in and around the p53-enriched region in the *Pax2* promoter.** **A)** ^32^P-labelled oligonucleotides (Table 1) were incubated with recombinant, purified C terminus-truncated and constitutively active p53. C, p53 consensus sequence ([[52](#_ENREF_52)]). Free probe and DNA-p53 complexes are indicated. **B)** Competition by p53 binding sites identified in Pax2 promoter with the p53 consensus sequence. Labeled consensus oligoduplex was incubated with p53 alone (lane 1) or in presence of unlabelled competitor oligoduplexes as described in Suppl. Methods. Diminished consensus-p53 complex indicates effective competition. **C)** Inhibition of complex formation between p53 and consensus binding site by addition of various unlabelled competitor oligoduplexes was quantified and plotted. Unlabelled consensus binding site and p21 promoter-p53 binding site compete effectively for p53 binding, whereas p53 binding sites from the Pax2 gene region (sites 1-14) show weak competition.

**Fig. S3. p53 binding sites are broadly scattered across the entire region including the intervening region between regions 1 and 2.** IGB view of Chromosome 19:44,830,572 - 44,836,404 is shown. a) Location of p53 binding motifs identified by Genomatix; b) Vertical green bars show p53 binding sites in p53-occupied region denoted by horizontal green bar; c) p53 ChIP-track; d) Input track; e) *Mus* Chromosome 19 coordinates.
